# Stress and Coping Strategies among Nursing Students in Clinical Practice during COVID-19

**DOI:** 10.3390/nursrep11030060

**Published:** 2021-08-11

**Authors:** Hanadi Y Hamadi, Nazik M. A. Zakari, Ebtesam Jibreel, Faisal N. AL Nami, Jamel A. S. Smida, Hedi H. Ben Haddad

**Affiliations:** 1 Brooks College of Health, University of North Florida, Jacksonville, FL 32224, USA; 2 College of Applied Sciences, Al Maarefa University, Riyadh 11597, Saudi Arabia; nzakari@mcst.edu.sa (N.M.A.Z.); fnalnami@kfmc.med.sa (F.N.A.N.); jsmida@mcst.edu.sa (J.A.S.S.); 3 Department of Finance and Investment, College of Economics and Administrative Sciences, Imam Mohammad Ibn Saud Islamic University, Riyadh 13318, Saudi Arabia; hhalhaddad@imamu.edu.sa

**Keywords:** COVID-19, nursing, students, clinical practice, stress, coping skills

## Abstract

Stress is common among nursing students and it has been exacerbated during the COVID-19 pandemic. This study examined nursing students’ stress levels and their coping strategies in clinical practice before and during the COVID-19 pandemic. A repeated-measures study design was used to examine the relationship between nursing students’ stress levels and coping strategies before and during the pandemic. Confirmatory factor analyses were conducted to validate the survey and a student T-test was used to compare the level of stress and coping strategies among 131 nursing students. The STROBE checklist was used. During COVID-19, there was a reliable and accurate relationship between stress and coping strategies. Furthermore, both stress and coping strategy scores were lower before COVID-19 and higher during COVID-19. Nursing students are struggling to achieve a healthy stress-coping strategy during the pandemic. There is a need for the introduction of stress management programs to help foster healthy coping skills. Students are important resources for our health system and society and will continue to be vital long term. It is now up to both nursing educators and health administrators to identify and implement the needed improvements in training and safety measures because they are essential for the health of the patient as well as future pandemics.

## 1. Introduction

Nursing is a practice-based profession, in the sense that the performance of nursing students depends largely on their clinical practicum; therefore, the quality of clinical training practice is crucial to the nursing education and profession. Furthermore, nursing students’ opinions regarding the quality of clinical training practices need to be strongly taken into consideration because of the demanding nature of the occupation. Nursing students are exposed to many sources of stress during clinical training and must handle stressful situations accordingly. Stressful situations can vary, including working with and handling breakout infections, where students assume an integral role in infection control measures and come into direct contact with infectious microorganisms. Becoming aware of and understanding students’ clinical practice stressors and coping strategies during clinical training in different situations provides educators with valuable information to maximize their students’ learning opportunities [1].

During a(n) pandemic/endemic, nursing students find themselves under additional stress factors such as the fear of being infected and infecting their close family members [2]. Two studies during the SARS (2003) and MERS outbreaks (2016) found that nursing students perceived themselves to be at a higher risk of infection and were reluctant to work in healthcare facilities due to inadequate safety and disease control measures [3,4]. Increased stress levels during the 2003 MERS outbreak in South Korea were negatively linked with nursing students’ intention to provide care to patients during future emerging infectious diseases [5].

Nursing students and staff are situated on the frontlines to combat infectious diseases and provide care and support to patients. They play a crucial role in providing effective infection control measures and ensuring the de-escalation of the spread of infectious microorganisms. Therefore, along with other medical staff and healthcare workers, nursing students and staff rushed to aid patients suffering from the most recent, fast-emerging, and rapidly spreading virus COVID-19 [6].

The COVID-19 pandemic spread to hospitals and nurses, putting them under enormous pressure in terms of workload and healthcare duties [7]. As a result, the lives and health of nurses and nursing students on the frontline, who are actively fighting the virus and are under great risk of contracting the disease, face dangerous repercussions [8]. COVID-19 studies and findings provide further evidence in regard to the anxiety experienced by nursing students and their response to treating this global pandemic [9].

Due to its extremely infectious and hazardous features, and the drastic lack of medication and treatment for the virus, COVID-19 has resulted in increased stress levels for nursing students and staff, which has consequently affected their coping strategies [8]. Therefore, understanding the relationship between stress levels and coping strategies of nursing students is critical. In non-pandemic times, the findings in Khater, Akhu-Zaheya [10], and Hamaideh [11] suggested that the most common coping behavior utilized by nursing students was problem-solving, followed by staying optimistic and transference. 

It is essential to evaluate the quality of the clinical practices and identify stressors that arise from different clinical settings according to nursing students’ perspectives. Therefore, this study aimed to examine nursing students’ stress levels and their coping strategies in clinical practice before and during the COVID-19 pandemic.

### Theoretical Framework

Stress has different definitions related to formulated theoretical models. It can be defined either as a stimulus, a response, or a combination of the two [12,13]. The definition of stress as a response was discovered by Selye (1976), who defines stress as the non-specific response of the body to any kind of demand [14,15]. On the other hand, Holmes and Rahes define stress as a stimulus without consideration to any response [16], stating that stress is: “an independent variable stimulus or load produced in an organism, creating discomfort, in such a way that whether tolerance limits are surpassed, stress becomes insufferable, appearing then psychological and physical problems”. 

The definition that is most relevant to and can be appropriately adopted in this study to explain the reality of nursing student’s stress during clinical practice is Lazarus and Folkman’s theoretical framework. Based on Lazarus’ theory regarding the difficulty in differentiating between response and stimulus as the definition of stress, he conceptualizes an apparent stress definition that can reconcile differences between the separate theories of stress as a response or stress as a stimulus. He defines stress as “A particular relationship between the person and the environment that is appraised by the person as taxing and/or exceeding his or her resources and endangering his or her well-being” [17]. This is because it describes stress as a transactional relationship between the person and their surrounding environment [17]. Stress is not a singular facet, but rather arises due to influencing factors that affect the individual and, in turn, impact their response in such situations. For example, one of these stressful situations can occur during students’ clinical practice once the students face a new environment and establish new relationships with staff nurses, patients, and an instructor and/or supervisor [18]. A study found that the most stressful clinical settings identified by the study were the intensive care unit followed by the emergency room, then the surgical units, while the area that was considered the least stressful was the medical units [19]. Therefore, this study uses this working definition of stress to examine nursing students’ stress levels and their coping strategies in clinical practice before and during the COVID-19 pandemic.

## 2. Materials and Methods

### 2.1. Setting

The study was conducted in the nursing department at a private University to evaluate and compare the students’ perspectives of clinical practice stressors and the coping strategies used to respond to these stressors before the COVID-19 pandemic and during the first wave of the COVID-19 pandemic. The findings from this study will be utilized to improve the learning and the educational process in their current situation, reflecting on the level of the students who will graduate from nursing school in the future.

### 2.2. Design and Sample

A repeated-measures study design was used. The sample nursing students were all undergraduate academic nursing students studying at a private University who are participating in clinical training. Students not in clinical training were excluded from the study. 

### 2.3. Data Collection Tool

This survey was developed using two previously validated surveys, the Perceived Stress Scale (PSS) and the Coping Behavior Inventory (CBI) survey. The PSS was developed by Sheu and Lin [20] and measures both the types of stressful events and the degree of stressors within clinical practices. This survey also included three demographic questions: The gender of the participant, their clinical training area, and their academic year of study. The PSS consists of 29 items (See Table 1) on a 5-point Likert scale (from 0 to 4) that are grouped into 6 stress/stressor categories. Those groups are stress from taking care of patients; teachers, and nursing personnel; assignments and workload; peers and daily life; the clinical environment; and lack of professional knowledge and skills. 

A score of 2.67 and higher was indicative of a high level of stress, a score between 1.34 and 2.66 was indicative of a moderate level of stress, and a score of less than 1.34 indicated a low level of stress [21]. The instrument’s reliability showed Cronbach’s alpha values of 0.86 and 0.89 [20,22] and a content validity index of 0.94 [22].

The CBI survey was first developed by Sheu and Lin [20] and measures the coping methods nursing students are more likely to utilize and their perceived effectiveness. The CBI survey consists of 19 items (See Table 1) all on a 5-point Likert Scale (from 0 to 4) that are grouped into 4 categories: Avoidance, Transference, Problem-solving, and Stay optimistic. A score of 2.67 and higher was indicative of a high level of coping strategies, a score between 1.34 and 2.66 was indicative of a moderate level of coping strategies, and a score of less than 1.34 indicated a low level of coping strategies. The instrument’s reliability showed a Cronbach’s alpha coefficient ranging from 0.76 to 0.80 [20,22].

### 2.4. Data Collection Procedure 

Prior to data collection, the study protocol was approved by the Institutional Review Board (IRB) of the university. A researcher approached all eligible nursing students at the end of in-person lectures and explained to them the purpose of the study. They were informed that participation in this study is voluntary, and they could withdraw from it at any time. A refusal to participate would not affect their learning process and academic results. Students who were interested in the study were asked to sign a paper or digital consent form, fill in the questionnaire, and immediately return it to the researcher. Other eligible students who did not have in-person lectures were sent the survey via a Google Form to invite them to participate and complete the survey. The survey was sent out to a total of 180 students. Nursing students completed the survey on paper and online between 1 January 2019, and 2 February 2019, for the period before COVID-19 and 30 September 2020, and 30 October 2020, for the period during COVID-19.

### 2.5. Participants 

Overall, 75 students were enrolled in clinical practice before and during COVID-19. One hundred and thirty-one nursing student responses were provided, resulting in about an 82% response rate before and during COVID-19. Out of the responses, 99 (75.6%) identified as female and 32 (24.4%) identified as male (See Table 2). The majority (60.3%) of the nursing students were in the Medical-Surgical clinical training area. In addition, 36 (27.5%) nursing students were in Level 5 (first year of clinical practice) of their academic year, and 32 (24.4%) were in Level 10 (last year of clinical practice also known as internship year) of their academic year. Nursing students in Level 5 participate in up to 2 clinical practice courses while Level 9 and 10 nursing students are in full clinical practice internships. The higher the level, the higher the clinical practice competency needed and the higher the necessary complexity. Only surveys that were fully completed were calculated in our response rate, therefore we had no missing data within the response for our analysis. 

### 2.6. Ethical Considerations

Before using the PSS and CBI tools, the researcher obtained permission from the original authors. The data collection tool contained a cover page that explained the aim of the study. All principles of ethics were adhered during the study. Therefore, anonymity and confidentiality of each individual’s data were also assured during the data collection stage. Participation in the survey was entirely optional and was at the discretion of each receiving the survey.

### 2.7. Statistical Analysis

The nursing student sample in this study was used to test the reliability and validity of the combined survey using confirmatory factor analysis. To analyze the results of the survey, means and standard deviations were utilized to examine the level of stress and coping strategies subscales and total scores. The Student T-test was used to compare the subscales and mean scores for the level of stress and coping strategies before and during the COVID-19 pandemic. We also used the Kolmogorov–Smirnov test to check the cumulative distributions of our two samples. All analyses were conducted in Stata 16, and significance was determined at *p* < 0.05. 

## 3. Results

The results from the comprehensive confirmatory factor analysis based on the varimax rotation factors of the entire sample results, the sample results before COVID-19, and the sample results after COVID-19 can be viewed in Table 2. Although not shown, the covariance between stress and coping strategies was positive and significant for all the sample (covariance = 0.4; *p* < 0.001), both the sample results before (covariance = 0.28; *p* < 0.001) and after (covariance = 0.58; *p* < 0.001) COVID-19. When examining the entire sample responses factor loading show in Table 3, all factor loadings were above 0.40 [23]. 

The overall average score of stress before COVID-19 was 1.32 (low stress) and 1.95 (moderate stress) during COVID-19 (See Table 4). Across all six stress categories, the average stress score was lower before COVID-19 than during COVID-19. The largest change was found in the stress category “lack of professional knowledge and skills” where the average stress score before COVID-19 was 0.95 (low stress) and 1.78 (moderate stress) during COVID-19 with a 0.83 change. The smallest change was found in the stress category “the environment” from an average stress level of 1.16 (low stress) before COVID-19 and 1.70 (moderate stress) during COVID-19. The overall average score of coping strategies before COVID-19 was 1.84 (moderate coping) and 2.17 (moderate coping) during COVID-19. Across all four coping strategies categories, the average coping strategies score is lower before COVID-19 than during COVID-19. The largest change was found in the coping strategy category “Transference” where the average coping strategy score before COVID-19 was 1.87 (moderate) and 12.41 (moderate) during COVID-19 with a 0.54 change. The smallest change was found in the coping strategy category “stay optimistic” from an average coping strategy level of 2.06 (low) before COVID-19 and 2.15 (moderate) during COVID-19. 

The results from the T-tests (See Table 4) show that there are statistically significant differences in both average stress scores and average coping strategies before and during COVID-19 across the majority of the categories. This statistical difference shows that both stress and coping strategy scores were lower before COVID-19 and higher during COVID-19. However, there was no statistically significant difference in the coping strategy category “Problem-solving” and “Stay optimistic” with a before-COVID-19 average coping strategy score of 2.09 and 2.06, and during scores of 2.32 and 2.15, respectively.

## 4. Discussion

Through the development of this survey, we have built upon previous research indicating the importance of understanding nursing students’ well-being through examining their stress levels and coping strategies. We have developed and tested a measurement scale that is reliable and accurately measures all identified in the Perceived Stress Scale (PSS) and the Coping Behavior Inventory (CBI) survey individually. However, our findings show that when the study is conducted on nurses in Saudi Arabia, there is not a strong reliable relationship between perceived stress and coping strategies (loading factor 0.4 and less) for the entire sample and the before COVID-19 sample. However, interestingly during COVID-19, there was a reliable and accurate relationship between stress and the use of coping strategies. A recent 2020 article regarding students’ coping strategies during the COVID-19 pandemic found that approximately 35% of students experienced some level of anxiety and used four types of coping strategies: Seeking social support, avoidance/acceptance, mental disengagement, and humanitarian [24].

The current study aimed to analyze the impact of the COVID-19 pandemic on nursing students’ stress levels and coping strategies. Through the combination of these two surveys, were have built upon previous research indicating the importance of stress and coping strategies among nursing students during unprecedented times. We have utilized a measurement scale that reliably and accurately measures stress and coping strategies before and during the COVID-19 pandemic. These findings can help inform nursing curricula developers on how to incorporate the needed skills and resources to prepare nurses for future infectious outbreaks. This is important as the Saudi Vision 2030 framework, released in 2017, has set a path to increase nurse graduates over the next 10 years and enhance the health delivery system to be community-focused. To meet this goal, Saudi Arabia has committed to increasing the nursing workforce by graduating and hiring 10,000 new nurses annually [25].

While multiple studies have reported on the psychological well-being of healthcare workers during COVID-19 [26,27,28,29,30,31,32,33,34], our study is one of the first to examine the influence of the pandemic by controlling for before the pandemic in nursing students in Saudi Arabia. Data collection occurred during the first wave of the pandemic in the country. The results of this study reflect an increased level of stress and coping strategies among nursing students during the continuing COVID-19 pandemic than before the pandemic. We found that, overall, across all subscales of stress there was a significant increase in stress relating to taking care of patients, teachers and nursing staff, assignments and workload, peers and daily life, lack of professional knowledge and skills, and the environment. These stressors can be attributed to multiple factors such as the unpreparedness to care for COVID-19 patients, increases in safety protocols in the clinical setting and decreases in safety personal protective equipment, relying heavily on simulation for training, and added assignments in an online learning environment to keep up with skill development. The stressful learning environment hinders student success. The completion of clinical practice and a precursor to licensure adds even more added pressure on students to complete an excessive workload to meet the non-direct care hours required [34]. 

According to previous research, even in normal circumstances, nursing students experience stress and must utilize several coping strategies to reduce both stress and anxiety. A study conducted in Bahrain found that almost all nursing students experience moderate to severe levels of stress while in their clinical practice [35]. Furthermore, another study found that over 99% of nursing students reported the level of perceived stress moderate or high. Several studies have revealed that the cause of clinical stress can be attributed to fear and uncertainty of unknown events, fear of medical errors, working with unfamiliar equipment, and gaps between theory and practice [36]. The additional increase in the level of stress among nursing students due to COVID-19 can have both internal and external consequences [37]. It can cause students to perform poorly and may lead to a withdraw from the program as self-doubt sets in, changes in mental and physical health, and can eventually affect the quality of care provided to patients. Several studies have shown that due to the demand and utilization of personal protective equipment across the globe, many direct care workers such as nurses and nursing students lacked the proper protective equipment, which increased their vulnerability to contracting COVID-19 [38,39]. As a result, many nurses have lost their lives to COVID-19, while others continue to fight against the deadly virus. Consequently, nurses perceive an increased risk of catching COVID-19 [40], which has increased turnover intentions [41]. However, a study conducted in China during the COVID-19 pandemic found that only 3% of their sample believed clinical nursing work to be “too dangerous to engage in” and have an increased intention of leaving the nursing profession [42].

The COVID-19 pandemic is currently the biggest threat to the lives and health of nurses and nursing students and has been shown to impact their emotional response and coping strategies. Our study shows that nursing students’ use of Avoidance and Transference as coping strategies and overall coping strategies increased during the COVID-19 pandemic in comparison to before the pandemic. However, our study did not identify a statistical difference between nursing students’ use of problem-solving or staying optimistic as coping strategies. This is in contradiction to a recent study that found that nursing students were more willing to use coping strategies that focused on problem-solving [8]. Our study findings can be explained by examining Gan and Liu's [43] study, which found that undergraduate students who regarded stressful events as controllable were more likely to apply problem-focused coping strategies; however, since COVID-related events were uncontrollable during the study period, students might have relied on emotion-focused coping strategies such as Avoidance and Transference, which contradict some priory studies [44,45]. A study conducted before the pandemic found that the most common coping behavior used by nursing students was transference, followed by staying optimistic and problem-solving, while the least used was Avoidance [46]. These findings are important for both nursing schools and hospitals, where they must focus on providing psychological support to nurses as well as training them in all available coping strategies to improve their ability to manage their emotions and effective coping tools to improve the lives of the nursing student, their families, and ultimately their patients. 

### Limitations

The study focused on nursing students in Saudi Arabia from a single private university. Due to the correlational nature of our study, no causal conclusions can be made; however, our findings may lead to a greater understanding of stress and coping strategies of nursing students involved in the COVID-19 pandemic. Hence, the findings should not be generalized to the overall student population.

## 5. Conclusions

The psychological impact of the pandemic on nursing students should not be ignored. The well-being of these students is affected by high levels of stress and emotional-based coping strategies. To alleviate the degree of impact, guidelines and strategies should be adopted into current nursing curricula even before the student is in clinical practice. Prioritizing research and policy effort on mental health, stress, and coping strategies of students needs to occur to equip future nursing students with the tools needed to be successful in the field of nursing. However, future research needs to replicate this study on a greater scale across multiple universities across multiple countries. Moreover, using in-depth data collection strategies, such as qualitative interviews or focus groups, in future research would significantly help explain the rationales behind why students adopted one coping strategies over another. 

### Relevance to Clinical Practice

Our study highlights that there was a strong, reliable, and accurate relationship between stress and the use of coping strategies during the COVID-19 pandemic compared to before. We anticipate that this relationship will only continue. Students are important resources for our health system and society and will continue to be vital long term. It is now up to both nursing educators and health administrators to identify and implement the needed improvements in training and safety measures because they are essential for the health of the patient, but also future pandemics.

## Figures and Tables

**Table 1:** The Perceived Stress Scale (PSS) and Coping Behavior Inventory (CBI) questions.

Subscales	Subscale Questions
Stress	
Stress from taking care of patients	Lack of experience and ability in providing nursing care and in making judgments Do not know how to help patients with physio-psycho-social problems Unable to reach one’s expectations Unable to provide responses to doctors’, teachers’, and patients’ questions Worry about not being trusted or accepted by patients or patients’ family Unable to provide patients with good nursing care Do not know how to communicate with patients Experience difficulties in changing from the role of student to that of a nurse
Stress from teachers and nursing staff	Experience discrepancy between theory and practice Do not know how to discuss patients’ illnesses with teachers, and medical and nursing personnel Feel stressed that teacher’s instruction is different from one’s expectations Medical personnel lack empathy and are not willing to help Feel that teachers do not give a fair evaluation on students Lack of care and guidance from teachers
Stress from assignments and workload	Worry about bad grades Experience pressure from the nature and quality of clinical practice Feel that one’s performance does not meet teachers’ expectations Feel that the requirements of clinical practice exceed one’s physical and emotional endurance Feel that dull and inflexible clinical practice affects one’s family and social life
Stress from peers and daily life	Experience competition from peers in school and clinical practice Feel pressure from teachers who evaluate students’ performance by comparison Feel that clinical practice affects one’s involvement in extracurricular activities Cannot get along with other peers in the group
Stress from lack of professional knowledge and skills	Unfamiliar with medical history and terms Unfamiliar with professional nursing skills Unfamiliar with patients’ diagnoses and treatments
Stress from the environment	Feel stressed in the hospital environment where clinical practice takes place Unfamiliar with the ward facilities Feel stressed from the rapid change in patient’s condition
Coping Strategy	
Avoidance	To avoid difficulties during clinical practice To avoid teachers To quarrel with others and lose temper To expect miracles so one does not have to face difficulties To expect others to solve the problem To attribute to fate
Problem-solving	To adopt different strategies to solve problems To set up objectives to solve problems To make plans, list priorities, and solve stressful events To find the meaning of stressful incidents To employ past experience to solve problems To have confidence in performing as well as senior schoolmates
Stay optimistic	To keep an optimistic and positive attitude in dealing with everything in life To see things objectively To have confidence in overcoming difficulties To cry, to feel moody, sad, and helpless
Transference	To feast and take a long sleep To save time for sleep and maintain good health to face stress To relax via TV, movies, a shower, or physical exercise (playing, jogging)

**Table 2:** Nursing student demographic characteristics, *n* = 131.

	Total		Before COVID-19 (*n* = 61)		During COVID-19 (*n* = 70)	
Variable	Frequency	Percent	Frequency	Percent	Frequency	Percent
Gender						
Male	32	24.4	8	13%	24	34%
Female	99	75.6	53	87%	46	66%
Clinical Training Area						
Medical-surgical	79	60.3	36	59%	43	61%
Critical care	16	12.2	7	11.5%	9	13%
Psychiatric	6	4.6	6	10%	0	0%
Maternity	12	9.2	7	11.5%	5	7%
all areas	18	13.7	5	8%	13	19%
Academic Year of Study						
Level 4	13	9.9	9	15%	4	6%
Level 5	36	27.5	12	20%	24	34%
Level 6	7	5.3	2	2%	5	7%
Level 7	10	7.6	7	11%	3	4%
Level 8	15	11.5	9	15%	6	9%
Level 9	18	13.7	6	10%	12	17%
Level 10	32	24.4	16	26%	16	23%

**Table 3:** Unstandardized estimated for all-sample, before and after COVID-19.

Measurement	All (*n* = 131)		Before COVID-19 (*n* = 61)		During COVID-19 (*n* = 70)	
	Standardized Factor Loading	mc^2^	Standardized Factor Loading	mc^2^	Standardized Factor Loading	mc^2^
Stress						
Stress from taking care of patients	0.79	0.62	0.83	0.68	0.69	0.47
Stress from teachers and nursing staff	0.86	0.74	0.84	0.71	0.84	0.70
Stress from assignments and workload	0.88	0.77	0.87	0.75	0.87	0.76
Stress from peers and daily life	0.87	0.77	0.83	0.79	0.89	0.79
Stress from lack of professional knowledge and skills	0.78	0.61	0.74	0.54	0.75	0.56
Stress from the environment	0.82	0.67	0.76	0.59	0.83	0.7
Coping Strategy						
Avoidance	0.59	0.35	0.62	0.38	0.53	0.28
Problem Solving	0.87	0.76	0.86	0.74	0.88	0.77
Stay optimistic	0.91	0.83	0.97	0.95	0.86	0.75
Transference	0.73	0.53	0.76	0.58	0.71	0.51
LR test	chi2(34)/df = 3.94		chi2(34)/df = 2.08		chi2(34)/df = 3.88	

Notes: LR test is the Wheaton et al. (1977) relative/normed chi-square (χ^2^/df), mc is the correlation between the dependent variable and its prediction, and mc^2^ = mc^2 is the Bentler-Raykov squared multiple correlation coefficient.

**Table 4:** Means and std. deviation and T-test for subscales items of stress experienced by nursing students and coping strategies in their clinical practice before and during the COVID-19 pandemic.

	Before COVID-19 (*n* = 61)			During COVID-19 (*n* = 70)			
Item	Mean	Std. Deviation	Levels	Mean	Std. Deviation	Levels	Sig.
Mean score of stress	1.32	0.80	low	1.95	0.76	moderate	0.000 *
Stress from taking care of patients	1.30	0.91	low	1.95	0.80	moderate	0.001 *
Stress from teachers and nursing staff	1.36	0.95	moderate	1.93	0.88	moderate	0.000 *
Stress from assignments and workload	1.59	0.94	moderate	2.21	0.85	moderate	0.000 *
Stress from peers and daily life	1.33	0.87	low	1.93	0.92	moderate	0.000 *
Stress from lack of professional knowledge and skills	0.95	0.94	low	1.78	1.03	moderate	0.004 *
Stress from the environment	1.16	1.01	low	1.70	1.09	Moderate	0.000 *
Mean score of coping strategies	1.84	0.85	moderate	2.17	0.75	moderate	0.019 *
Avoidance	1.47	0.89	moderate	1.90	0.91	moderate	0.007 *
Problem-solving	2.09	1.09	moderate	2.32	0.93	moderate	00.20
Stay optimistic	2.06	0.99	moderate	2.15	0.87	moderate	00.55
Transference	1.87	0.95	moderate	2.41	1.04	moderate	0.02 *

* *p*-value for Chi-squared test < 0.05.

## Data Availability

The data that support the findings of this study are available on request from the corresponding author. The data are not publicly available due to privacy or ethical restrictions.
